# Piperine Targets Different Drug Resistance Mechanisms in Human Ovarian Cancer Cell Lines Leading to Increased Sensitivity to Cytotoxic Drugs

**DOI:** 10.3390/ijms22084243

**Published:** 2021-04-19

**Authors:** Karolina Wojtowicz, Karolina Sterzyńska, Monika Świerczewska, Michał Nowicki, Maciej Zabel, Radosław Januchowski

**Affiliations:** 1 Department of Histology and Embryology, Poznań University of Medical Sciences, Święcickiego 6 St., 61-781 Poznań, Poland; k.olejniczak@ump.edu.pl (K.S.); mswierczewska@ump.edu.pl (M.Ś.); mnowicki@ump.edu.pl (M.N.); 2 Department of Anatomy and Histology, Collegium Medicum, University of Zielona Gora, Zyty 28 St., 65-046 Zielona Gora, Poland; m.zabel@cm.uz.zgora.pl; 3 Division of Histology and Embryology, Department of Human Morphology and Embryology, Wroclaw Medical University, T. Chałubińskiego 6a St., 50-368 Wroclaw, Poland

**Keywords:** ovarian cancer, piperine, drug resistance, protein phosphorylation, drug transporters, cancer stem cells, cell adhesion-mediated drug resistance, extracellular matrix

## Abstract

Our goal was to examine the anticancer effects of piperine against the resistant human ovarian cancer cells and to explore the molecular mechanisms responsible for its anticancer effects. Our study used drug-sensitive ovarian cancer cell line W1 and its sublines resistant to paclitaxel (PAC) and topotecan (TOP). We analyzed the cytotoxic effect of piperine and cytostatic drugs using an MTT assay. The impact of piperine on protein expression was determined by immunofluorescence and Western blot. We also examined its effect on cell proliferation and migration. We noticed a different level of piperine resistance between cell lines. Piperine increases the cytotoxic effect of PAC and TOP in drug-resistant cells. We observed an increase in PTPRK expression correlated with decreased pTYR level after piperine treatment and downregulation of P-gp and BCRP expression. We also noted a decrease in COL3A1 and TGFBI expression in investigated cell lines and increased COL3A1 expression in media from W1PR2 cells. The expression of Ki67 protein and cell proliferation rate decreased after piperine treatment. Piperine markedly inhibited W1TR cell migration. Piperine can be considered a potential anticancer agent that can increase chemotherapy effectiveness in cancer patients.

## 1. Introduction

Ovarian cancer is a heterogeneous malignancy with variable clinical development that remains the most challenging disease in gynecologic oncology [1,2]. Most ovarian cancer patients are diagnosed in advanced stages (III or IV according to FIGO classification). The prognosis of ovarian cancer is directly related to the stage of tumor and tumor cells remaining after resection [3]. Surgical resection along with platinum-based chemotherapy is a standard treatment option for ovarian cancer. After surgery, patients undergo the platinum/taxane treatment as the first-line chemotherapeutic modality [4,5]. About 5% of patients are primarily resistant to platinum with a lack of remission or progression during treatment. The others can be divided into the following groups: not sensitive to platinum—recurrence within six months after treatment (about 17%), partially sensitive to platinum—recurrence within 6–12 months after completion of treatment (about 23%) and sensitive to platinum—recurrence after 12 months or more (about 55%). However among patients sensitive at the beginning of treatment, only about 18% is probably cured (120 months without recurrence). Others develops progression within 12–60 months after treatment (about 33%) or within 60–120 months after treatment (about 4%) [6,7]. The second-line combinational therapy with platinum and other drugs is beneficial for patients with partially sensitive or sensitive tumors with recurrence after 6–12 months or more [8]. In the case of platinum-resistant tumors, drugs such as topotecan (TOP), doxorubicin (DOX), and gemcitabine are implemented [9,10]. Unfortunately for most drugs, the response to second-line chemotherapy amounts to 15–35%.

Unsuccessful ovarian cancer chemotherapeutic treatment results from primary drug resistance or that developed during treatment. Multidrug resistance (MDR) is a phenotype whereby cancer cells acquire cross-resistance to various compounds and, it is frequently observed in ovarian cancer. The most important proteins responsible for MDR are drug transporters from the ABC family, and among them, the leading players are glycoprotein P (P-gp) and breast cancer resistant protein (BCRP) [11]. Increased P-gp and BCRP expression is associated with decreased progression-free survival in ovarian cancers [12]. We also observed increased P-gp protein expression in PAC and DOX-resistant and BCRP overexpression in TOP-resistant ovarian cancer cell lines [13].

The interaction between the drug and the tumor microenvironment is another factor that may influence drug resistance development [14]. The dense cellular structure in the tumor and increased ECM components′ expression by cancer cells and tumor-associated fibroblasts may limit drug diffusion [15,16]. Additionally, drugs such as methotrexate (MTX), DOX, and PAC can be directly bound by ECM molecules that block their availability to the tumor cells [17]. ECM components can also play an active role in drug resistance. The binding of cancer cells to ECM activates the intracellular signals transduction and is designated as a cell adhesion-mediated drug resistance (CAM-DR) [18,19]. However, the expression of ECM components and CAM-DR seems not to be limited to cancer tissue. Recently, we [20,21,22,23] and others [24] observed the expression of different ECM components in drug-resistant cancer cell lines suggesting their active role in CAM-DR [25,26]. Notably, we observed a very high expression of COL3A1 in PAC and TOP-resistant cell lines suggesting the role of this protein in resistance to these drugs [27,28]. Furthermore, we observed different cell populations with low and very high COL3A1 expression and the presence of extracellular COL3A1 in cell culture [27]. According to the ECM-mediated drug resistance development model, cancer cells with a high expression level of ECM molecules are more resistant than other cells in the tumor and survive chemotherapy [19].

The poor survival and prognosis of individuals with cancer are often due to the presence of cancer stem cells (CSCs) [29]. CSCs are inherently resistant to radio- and chemotherapy [30]. A hallmark of CSCs is that the cells robustly express drug transporters on the cell surface, thus exhibiting a multi-drug resistance (MDR) phenotype [31]. High ALDH activity is a unique feature for CSCs that distinguishes them from other cancer cells in the tumor [32]. The expression of ALDH isoform 1A1 (ALDH1A1) is considered the universal marker of cancer cells among solid tumors [33]. According to the CSCs model and our results, ALDH1A1-positive cancer cells can be responsible for drug resistance development in ovarian cancer [34].

Recently, we observed that ALDH1A1-positive CSCs express a higher level of ECM proteins than other cells [23,27]. Thus, we created our own drug resistance model designated as a CSCs/ECM model of drug resistance. According to this model, CSCs with a high ECM expression level survive chemotherapy and then divide and repopulate the tumor mass. After therapy, the content of CSCs/ECM cells in the tumor mass increases, and all of them are resistant because of the high level of drug transporters expression and ECM expression [23].

Increased drug resistance also seems to be related to increased protein phosphorylation and stronger signal transduction [35]. Increased protein phosphorylation was associated with drug resistance development in cancer [36]. Changes in protein phosphorylation can be related both to increased expression/activity of kinases and decreased expression/activity of protein phosphatases [37]. Reduced expression of Protein Tyrosine Phosphatase Receptor Type K was associated with poor prognosis in breast cancer [38] and decreased activity of PTPRK with less resistant phenotype in gliomas [39]. We recently observed reduced expression of PTPRK and increased level of total tyrosine phosphorylation in fifteen drug resistant ovarian cancer cell lines [40].

Thus, drug resistance of the cancer cells is a very complex phenomenon and is a challenge in the field of cancer chemotherapy. Nowadays, despite many efforts, chemotherapy is not fully effective. Scientists are looking for new factors that could be used as drugs in cancer treatment. More and more attention is put on naturally occurring substances [41,42,43,44]. Phytochemicals derived from spices, including peppers, are considered important for developing potential antitumor agents [45]. Piperine is isolated from long and black peppers species such as *Piper longum* and *Piper nigrum* [41]. Several of the piperine bioactivities have been reported, including insecticidal, anti-inflammatory, anti-bacterial and anticancer [46]. The cytotoxic effect of piperine has been reported in the A2780 ovarian cancer cell line [45]. Furthermore, piperine also increases the cytotoxic effect of PAC in breast and ovarian cancer cell lines [47,48,49]. Another study showed that piperine increases the effect of cytotoxic drugs by decreasing the expression of drug transporters and their activity in vitro and in vivo [50,51]. The decrease in the number of ALDH1+ cells in breast cancer cell line after piperine treatment has been also reported which implies targeting CSCs signaling pathways by piperine [52]. Piperine exerted antiproliferative effects on the OVCAR-3 ovarian cancer cells. It was concomitant with the upregulation of apoptotic proteins such as caspase 3 and 9 and Bax expressions. It is believed that piperine also induced the cells’ arrest in the G2/M phase of the cell cycle [53]. Moreover, piperine affects diverse signaling pathways associated with cancer cell growth and survival, including mitogen-activated protein kinase (MAPK), PI3K/Akt, and STAT3 pathways [47,54]. It suppresses tumor cell metastasis in gastric cancer, represses cell proliferation and migration, and promotes apoptosis in prostate cancer cells [55].

To study piperine’s ability to breakdown chemotherapy resistance in ovarian cancer, we used a well-characterized drug resistance development model composed of drug-sensitive ovarian cancer cell line W1 and its sublines resistant to PAC (W1PR1, W1PR2) and TOP (W1TR). All the cell lines were previously characterized according to drug cross-resistance [13], expression of drug transporters [13], presence of ALDH1A1 CSCs [23,34], expression of ECM molecules, especially collagens [27,28], and the level of pTYR [40]. Our findings suggest that piperine targets different drug resistance mechanisms and may potentially be a therapeutic agent for preventing and treating ovarian cancer. 

## 2. Results

### 2.1. Cell Survival Assay

**Figure 1:**
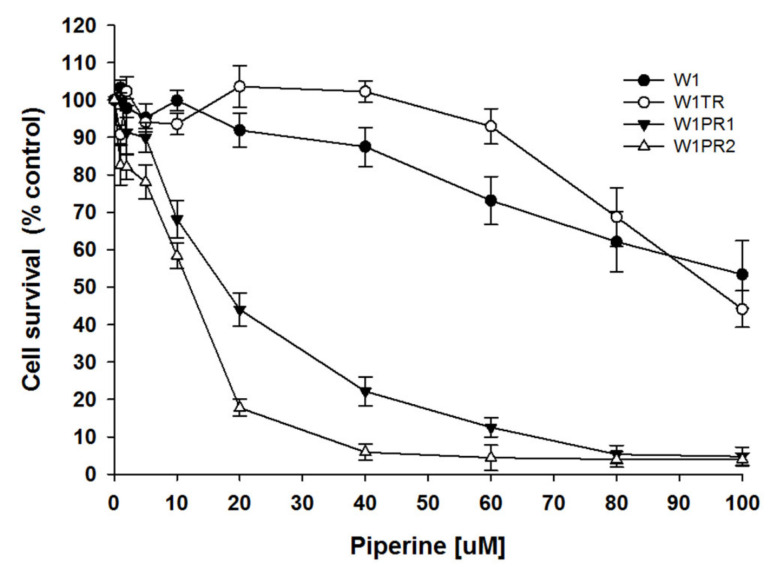
MTT cell survival assay for W1, W1TR, W1PR1, and W1PR2 cell lines treated with or without increasing piperine concentrations. The cell viability assay was expressed as a percent of untreated control (mean ± SEM).

In the first step of our study, we used MTT assay to check piperine’s cytotoxic effect in investigated cell lines. Figure 1 shows cell survival for W1, W1TR, W1PR1, and W1PR2 cell lines treated with piperine in the range of 0 to 100 µM. TOP-resistant cell line W1TR showed similar sensitivity to piperine similar to parental drug-sensitive cell line W1. In contrast, both cell lines resistant to PAC were more susceptible to the piperine treatment. The appointed IC25 and IC50 values for all cell lines are summarized in Table 1. We did not observe statistically significant differences in IC25 and IC50 values between W1 and TOP-resistant cell line. However, both PAC-resistant cell lines showed a statistically significant decrease in IC25 and IC50 values than the parental W1 cell line (*p* < 0.01).

**Figure 2:**
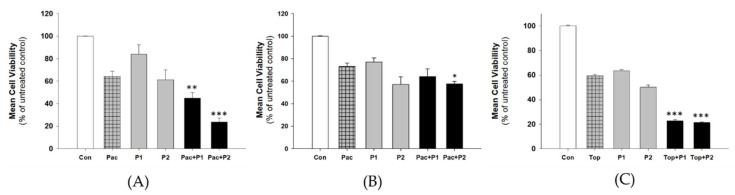
Piperine sensitizes drug-resistant cell lines to chemotherapy in vitro. Cell lines resistant to PAC–W1PR1 (**A**) and W1PR2 (**B**) and TOP (**C**) were seeded in 96-well plates. Cells were treated for 72 h with PAC (500 ng/mL = 0.59 µM) or TOP (50 ng/mL = 0.11 µM), piperine in concentrations P1 and P2, or with PAC/TOP and piperine together. After 72 h of treatment, cell viability was determined using MTT assay. Viability was expressed as a percent of the untreated control (mean ± SEM). Values were considered significant at * *p* < 0.05, ** *p* < 0.01 and *** *p* < 0.001.

Next, we were interested in determining whether piperine might increase PAC and TOP-induced cytotoxic effects in our investigated cell lines The diagrams A, B, and C of Figure 2 shows the mean of cell viability for the cell lines treated with the drug (PAC or TOP), piperine in two different concentration P1 and P2, and the effect of both components simultaneously, as indicated by PAC+P1, PAC+P2, TOP+P1, TOP+P2. We used cytotoxic drugs at concentrations that decrease cell viability to 60–70%. We noticed that at both concentrations, used piperine statistically significantly increased the cytotoxic effect of PAC in the W1PR1 cell line (*p* < 0.01 for P1 and *p* < 0.001 for P2 concentration, respectively) (Figure 2A). A similar effect was observed for the W1TR cells (Figure 2C); the changes were significant at *p* < 0.001. In the case of the W1PR2 cell line (Figure 2B), the significant decrease in cell viability (*p* < 0.05) was noted only at higher piperine concentration.

### 2.2. Immunofluorescence Analysis of PTPRK and pTYR after Piperine Treatment

**Figure 3:**
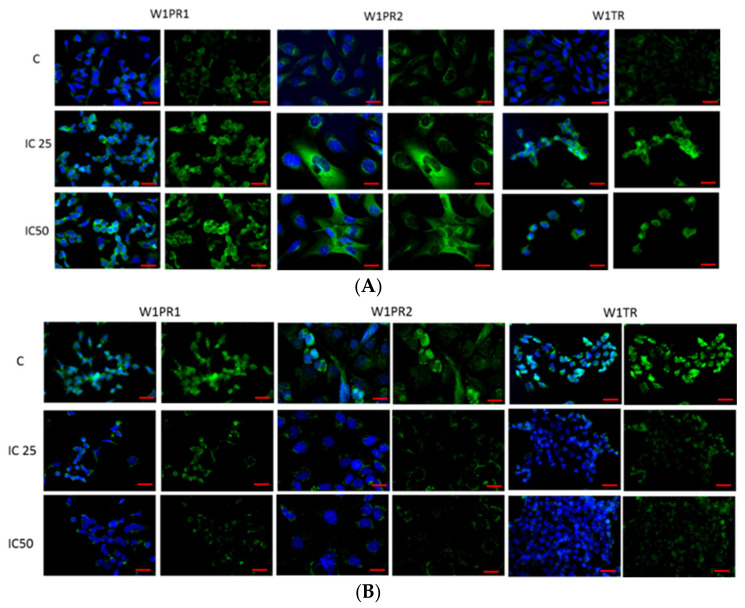
Immunofluorescence visualization of PTPRK (**A**) and pTyr (**B**) expression in PAC and TOP-resistant cell lines after piperine treatment. PTPRK was detected using the anti-PTPRK antibody and an Alexa Fluor^®^488-conjugated secondary antibody (green). pTyr was detected using the anti-pTyr antibody and MFP488-conjugated secondary antibody (green). Cell nuclei were stained with DAPI (blue). Row C shows the control state (cells without piperine), IC25 and IC50 show the cells treated with piperine for 72 h. The first column shows both DAPI (blue) and target protein signal (green), the second column shows the target protein signal alone. Scale bar = 2-µm.

Since previously, we observed decreased PTPRK expression in W1PR1, W1PR2, and W1TR cell lines [40], we were interested if sensitization of cell lines to cytotoxic drugs can be related to changes in PTPRK expression and protein phosphorylation. Thus, we performed fluorescence analysis of PTPRK and pTYR expression after piperine treatment in investigated cell lines. We observed that piperine caused, in both IC25 and IC50 concentrations, an increase in PTPRK expression for all tested cell lines (Figure 3A). Furthermore, increased PTPRK expression correlated with decreased pTYR level after piperine treatment (Figure 3B).

### 2.3. Analysis of ALDH1A1 Expression after Piperine Treatment

**Figure 4:**
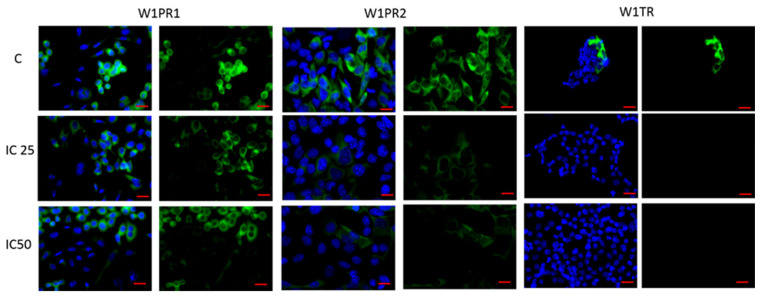
Immunofluorescence visualization of ALDH1A1 expression in PAC and TOP resistant cell lines after piperine treatment. ALDH1A1 was detected using the anti-ALDH1A1 antibody and an Alexa Fluor^®^488-conjugated secondary antibody (green). Cell nuclei were stained with DAPI (blue). Row C shows the control state (cells without piperine), IC25 and IC50 show the cells treated with piperine for 72 h. The first column shows both DAPI (blue) and target protein signal (green), the second column shows the target protein signal alone. Scale bar = 2-µm.

As ALDH1A1 is a universal marker of CSCs [33] and piperine has been described to target ALDH1A1+ cancer stem cells [52], we were interested in whether piperine can influence ALDH1A1 expression in investigated cell lines. As previously [23,27,34,40], immunofluorescence experiments confirmed the presence of ALDH1A1+ and ALDH1A1- cells in all analyzed cell lines. After piperine treatment in the W1PR1 cells, we observed a low decrease in ALDH1A1 expression. Meanwhile, the fluorescence intensity of this protein decreases sharply in W1PR2 and W1TR cells after piperine treatment (Figure 4). However, a difference between these cell lines was observed. In the W1TR cell line, we observed a total loss of ALDH1A1 expression. In the W1PR2 cell line, we still observed ALDH1A1+ cells, although the fluorescence signal is very low.

**Figure 5:**
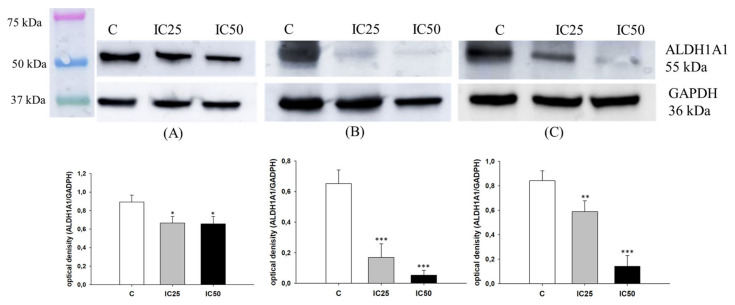
ALDH1A1 protein expression analysis in the W1PR1 (**A**), W1PR2 (**B**), and W1TR cell lines (**C**). Protein lysates were prepared after 72 h of cell culture at piperine concentrations at the IC25 or IC50 along with the control untreated cells (**C**). The cellular proteins were separated using 7% PAGE and transferred to a PVDF membrane, which was then immunoblotted with either primary Ab or HRP-conjugated secondary Ab. A primary anti-GADPH Ab was used as a loading control for the cell lysates. The graphs show the results of the densitometry quantification of the Western blot analysis optical density, which is presented as an ALDH1A1/GADPH ratio. The values were considered significant at * *p* < 0.05, ** *p* < 0.01, and *** *p* < 0.001.

Next, we analyzed ALDH1A1 protein expression in all resistant cell lines by Western blot (Figure 5). In the W1PR1 cell line, a low decrease in ALDH1A1 expression after piperine treatment (*p* < 0.05) was observed, corresponding to a lower fluorescence signal (Figure 5A). However, the most noticeable effect was observed in W1PR2 (*p* < 0.001 for IC25 and IC50) and W1TR cell lines (*p* < 0.01 for IC25 and *p* < 0.001 for IC50), where we observed a strong dose-dependent decrease in ALDH1A1 expression (Figure 5B,C).

### 2.4. Analysis of MDR Proteins Expression after Piperine Treatment

**Figure 6:**
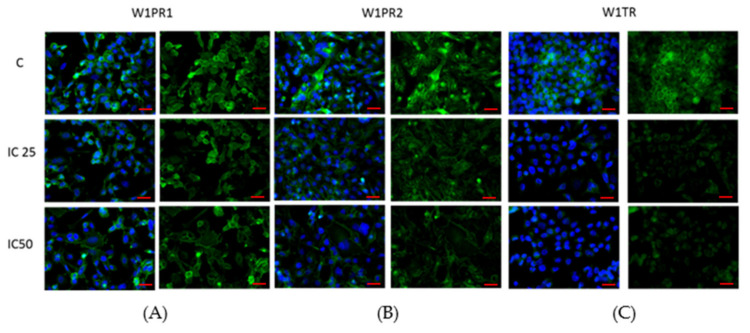
Immunofluorescence visualization of P-gp (**A**,**B**) and BCRP (**C**) expression in PAC and TOP-resistant cell lines after piperine treatment. P-gp was detected using the anti-P-gp antibody and an Alexa Fluor^®^488-conjugated secondary antibody (green). BCRP was detected using the anti-BCRP antibody and MFP488-conjugated secondary antibody (green). Cell nuclei were stained with DAPI (blue). Row (**C**) shows the control state (cells without piperine), IC25 and IC50 show the cells treated with piperine for 72 h. The first column shows both DAPI (blue) and target protein signal (green), the second column shows the target protein signal alone. Scale bar = 2-µm.

**Figure 7:**
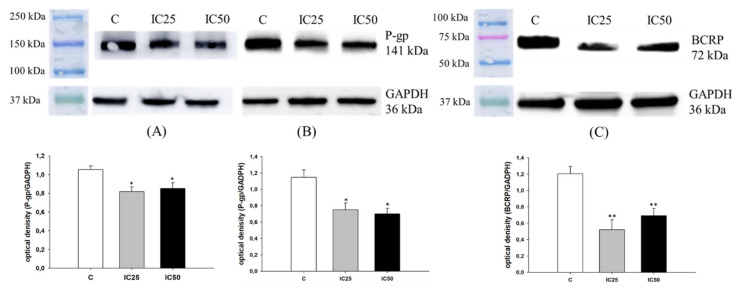
P-gp protein expression analysis in the W1PR1 (**A**), W1PR2 (**B**), and BCRP protein expression analysis W1TR cell line (**C**). Protein lysates were prepared after 72 h of cell culture at piperine concentrations at the IC25 or IC50 along with the control untreated cells (**C**). The cellular proteins were separated using 7% PAGE and transferred to a PVDF membrane, which was then immunoblotted with either primary Ab or HRP-conjugated secondary Ab. A primary anti-GADPH Ab was used as a loading control for the cell lysates. The graphs show the results of the densitometry quantification of the Western blot analysis optical density, which is presented as a P-gp/GADPH (**A**,**B**) or BCRP/GADPH (**C**) ratio. The values were considered significant at * *p* < 0.05 and ** *p* < 0.01.

The primary mechanism of drug resistance in investigated cell lines seems to be related to a very high expression level of P-gp in PAC-resistant cell lines and BCRP expression in TOP-resistant cell lines [13]. Therefore, we were interested if piperine can change the expression of these drug transporters. Immunofluorescence visualization of P-gp shows a minimal decrease in fluorescence signal after piperine treatment in the W1PR1 cell line and a low decrease in the W1PR2 cell line (Figure 6). However, a strong decrease in BCRP fluorescence was detected for IC25 and IC50 piperine concentrations (Figure 6). We also checked the influence of piperine on MDR proteins expression (Figure 7). We noted a small decrease in P-gp expression in W1PR1 (*p* < 0.05) and W1PR2 cells (*p* < 0.05) (Figure 7A,B). However, no difference in MDR expression between piperine IC25 and IC50 was noted. In the W1TR cell line, we observed a downregulation of BCRP expression after piperine treatment (*p* < 0.01) (Figure 7C).

### 2.5. Analysis of COL3A1 Expression after Piperine Treatment

**Figure 8:**
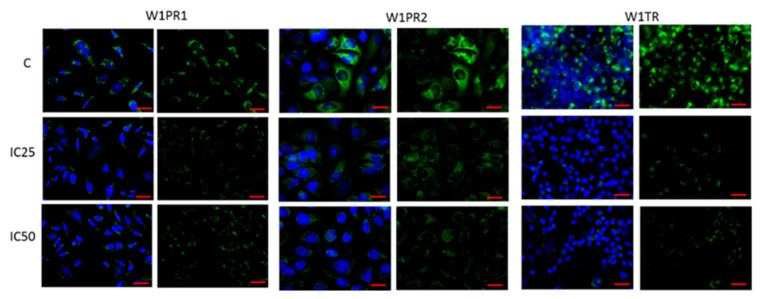
Immunofluorescence visualization of COL3A1 expression in PAC and TOP-resistant cell lines after piperine treatment. COL3A1 was detected using the anti-COL3A1 antibody and an Alexa Fluor^®^488-conjugated secondary antibody (green). Cell nuclei were stained with DAPI (blue). Row C shows the control state (cells without piperine), IC25 and IC50 show the cells treated with piperine for 72 h. The first column shows both DAPI (blue) and target protein signal (green), the second column shows the target protein signal alone. Scale bar = 2-µm.

**Figure 9:**
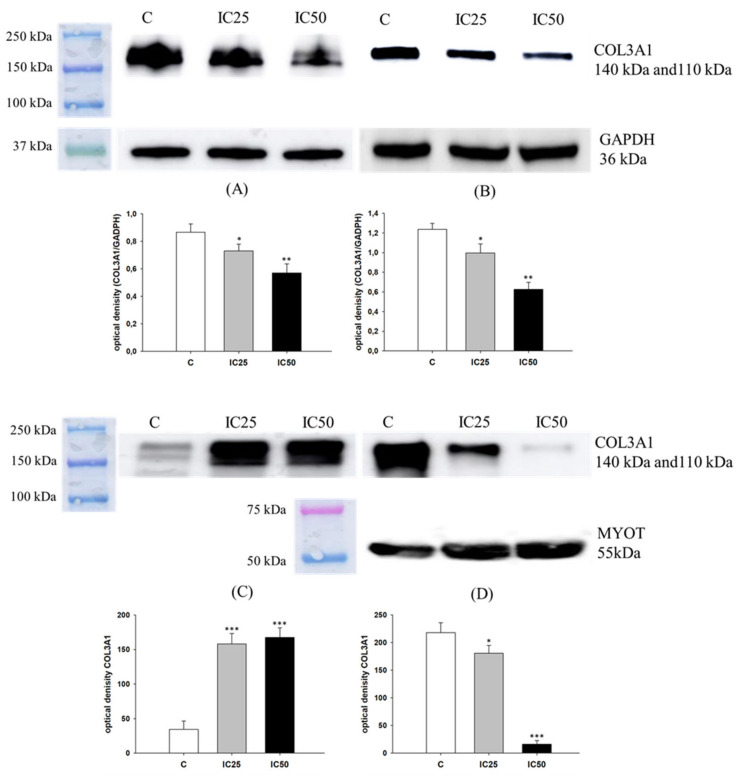
COL3A1 protein expression analysis in the W1PR2 (**A**) and W1TR cell lines (**B**) and their corresponding media (**C**,**D**), respectively. Protein lysates were prepared after 72 h of cell culture at piperine concentrations at the IC25 or IC50 along with the control untreated cells (C). Proteins were separated using 7% PAGE and transferred to a PVDF membrane, which was then immunoblotted with either primary Ab or HRP-conjugated secondary Ab. A primary anti-GADPH Ab was used as a loading control for the cell lysates. MYOT was used as a loading control for medium from W1TR cell line. The graphs show the results of the densitometry quantification of the Western blot analysis optical density, which is presented as a COL3A1/GADPH ratio (**A**,**B**) or COL3A1 optical density (**C**,**D**). The values were considered significant at * *p* < 0.05 and ** *p* < 0.01, *** *p* < 0.001.

Previously, we observed a moderate increase in COL3A1 expression in W1PR1 cell line and a very high increase in COL3A1 expression in W1PR2 and W1TR cell lines compared to the parental drug-sensitive W1 cell line, suggesting its potential significance in drug resistance [27,28]. Thus, we were interested if piperine treatment can affect COL3A1 expression. We observed a low fluorescence signal of COL3A1 in the W1PR1 cell line and a decrease in COL3A1 expression after piperine treatment in all cells (Figure 8). Following our previous observation in the W1PR2 and W1TR cell lines, we observed cells with a low and very high level of COL3A1 expression [23,27,28]. After piperine treatment, we noted a decrease in fluorescence intensity compared to the control, but it seems that piperine affects mainly cells with high level of COL3A1 expression (Figure 8). We also performed the analysis of COL3A1 protein after piperine treatment. In W1PR1 cell lines, we could not detect COL3A1 expression using Western blot (not shown). We observed two bands corresponding to two COL3A1 splicing variants with a molecular mass of about 140 kDa and 110 kDa in the W1PR2 cell line (in cell lysates and cell culture medium) (Figure 9A,C) and in cell culture medium from the W1TR cell line (Figure 9D). In lysates from the W1TR cell line, we noted only one band corresponding to a molecular mass of 140 kDa (Figure 9B), which is consistent with our previous observation [27]. A decrease in COL3A1 expression in W1PR2 and W1TR cells for both piperine concentration IC25 (*p* < 0.05) and IC50 (*p* < 0.01) (Figure 9A,B) was observed. However, we noted a more visible effect for piperine IC50 concentration. We also checked if piperine can affect extracellular collagen levels that previously we observed secreted COL3A1 in both cell lines [27,28]. In corresponding media, the COL3A1 expression significantly increased in W1PR2 cells (*p* < 0.001) (Figure 9C)—in contrast to the W1TR cell line—we noted lower COL3A1 expression for IC25 (*p* < 0.05) and almost a lack of expression for IC50 (*p* < 0.001) (Figure 9D). Previously we observed a high expression level of myotilin in the W1TR cell line; thus, the corresponding medium [21] was used here as a loading control for medium from the W1TR cell line.

### 2.6. Analysis of TGFBI Expression after Piperine Treatment

**Figure 10:**
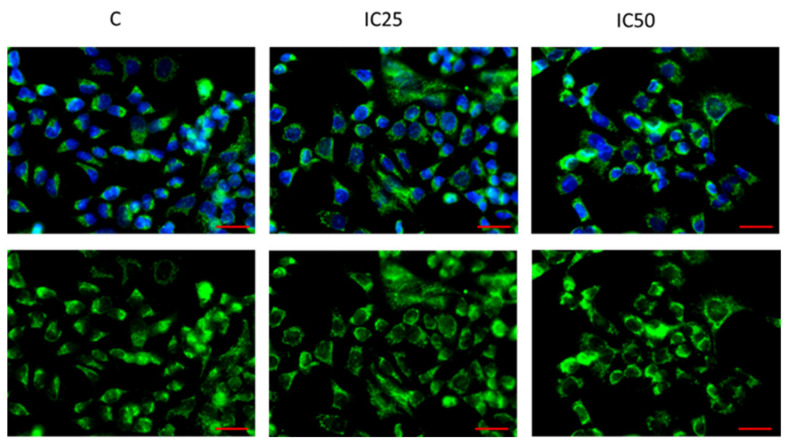
Immunofluorescence visualization of TGFBI expression in TOP-resistant cell line after piperine treatment. TGFBI was detected using the anti-TGFBI antibody and an Alexa Fluor^®^488-conjugated secondary antibody (green). Cell nuclei were stained with DAPI (blue). Column C shows the control state (cells without piperine); IC25 and IC50 show the cells treated with piperine for 72 h. The first row shows both DAPI (blue) and target protein signal (green), the second row shows the target protein signal alone. Scale bar = 2-µm.

**Figure 11:**
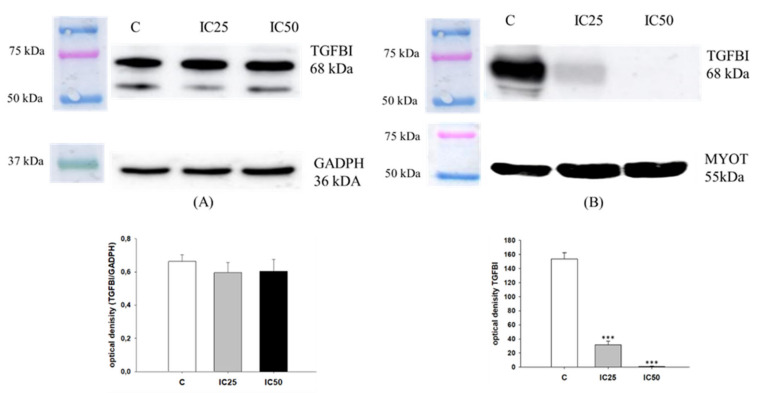
TGFBI protein expression analysis in the W1TR cell line (**A**) and corresponding media (**B**). Protein lysates were prepared after 72 h of cell culture at piperine concentrations at the IC25 or IC50 along with the control untreated cells (C). Proteins were separated using 7% PAGE and transferred to a PVDF membrane, which was then immunoblotted with either primary Ab or HRP-conjugated secondary Ab. A primary anti-GADPH Ab was used as a loading control for the cell lysates. MYOT was used as a loading control for medium from W1TR cell line. The graphs show the results of the densitometry quantification of the Western blot analysis optical density, which is presented as a TGFBI/GADPH ratio (**A**) or TGFBI optical density (**B**). The values were considered significant at *** *p* < 0.001.

Previously, we reported an increased expression of TGFBI in three TOP-resistant ovarian cancer cell lines [56]. Here, we investigated if piperine treatment can change TGFBI expression in the W1TR cell line. To determine TGFBI protein expression in the W1TR cell line, we performed fluorescence analysis before and after piperine treatment. No changes in fluorescence signal after piperine treatment were observed in this experiment (Figure 10). In the next step, we tested the effect of piperine on TGFBI protein expression in W1TR cells and corresponding media (Figure 11). We observed no difference in TGFBI expression in W1TR cells before and after piperine treatment (Figure 11A). However, in corresponding media, we noted a decrease in TGFBI expression according to the increasing piperine concentration (*p* < 0.001). No differences in MYOT protein after piperine treatment were observed (Figure 11B).

### 2.7. Effect of Piperine on Cell Proliferation

**Figure 12:**
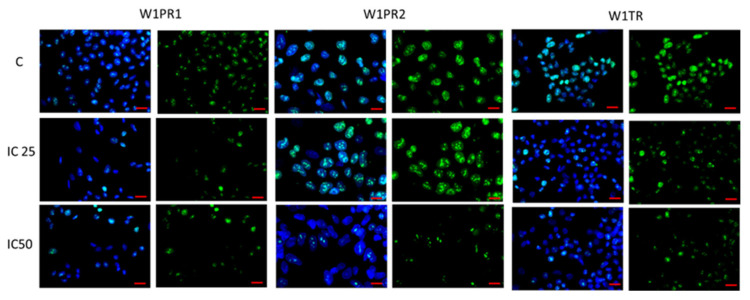
Immunofluorescence visualization of Ki67 expression in PAC and TOP-resistant cell lines after piperine treatment. Ki67 was detected using the anti-Ki67 antibody and an Alexa Fluor^®^488-conjugated secondary antibody (green). Cell nuclei were stained with DAPI (blue). C row means control (cells without piperine), IC25 and IC50 are showing the cells treated in appropriate concentration of piperine for 72 h. The first column shows both DAPI (blue) and protein signal (green), the second column shows protein signal alone, and so for each cell line. Scale bar = 2-µm.

To determine the effect of on cell proliferation, the Ki67 protein expression was investigated at the cellular level in the immunofluorescence experiment. We detected fluorescence signal in nuclei of drug-resistant cell lines W1PR1, W1PR2 and W1TR. In those cells, the expression of Ki67 protein decreased after piperine treatment in IC50 concentration (Figure 12). However, the difference was present in IC25 concentration. An apparent decrease in the number of positive cells was observed in W1PR1 and W1TR cell lines. In contrast, no difference was observed in the W1PR2 cell line (Figure 12).

**Figure 13:**
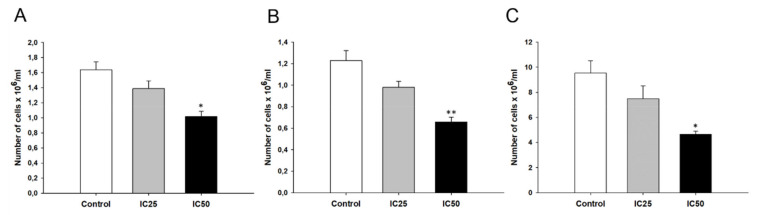
Cells proliferation after piperine treatment in W1PR1 (**A**), W1PR2 (**B**), and W1TR (**C**) cell lines. For each cell line the control is shown, and results obtained for piperine IC25 and IC50 concentrations. Bars represent the mean number of cells after 72 h incubation ± SD values. * *p* < 0.05; ** *p* < 0.01; * Represents the comparison with the control group.

Incubation of W1PR1, W1PR2, and W1TR cells with piperine (at IC25 and IC50 concentration) inhibited cellular proliferation compared to control cells. The experiment demonstrated an inhibitory effect at a statistically significant level only when piperine was used in a concentration of IC50 (Figure 13).

### 2.8. Effect of Piperine on Cells Migration

**Figure 14:**
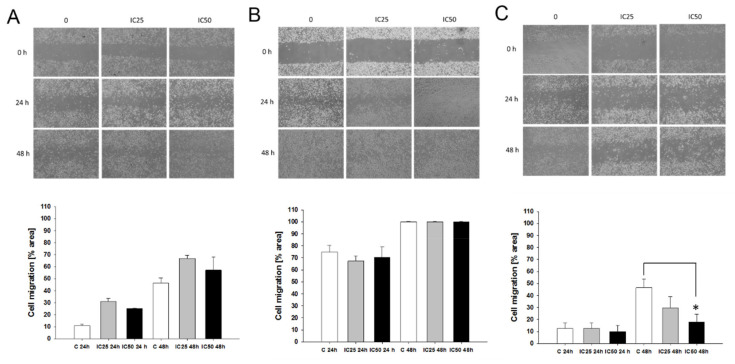
Effect of piperine on the migration of the W1PR1 (**A**), W1PR2 (**B**), and W1TR (**C**) cells. Cell monolayers were scratched with a pipette tip and pretreated with control C and piperine in IC25 and IC50 concentration for 24 h and 48 h. Migrating cells were visualized and photographed by phase-contrast microscopy. The experiments were performed in triplicate. Bars represent the mean of cell migration ± SD values. * *p* < 0.05.

To determine the effect of piperine on cell migration inhibition, we performed an in vitro model of cell injury. In this wound-healing assay, recovery of the wounded area was measured 24 and 48 h following the scratch (Figure 14). Compared to control cells, piperine markedly inhibited W1TR cell migration into the wounded area (Figure 14C). This effect was mainly observed after 48 h and when piperine was used in IC50 concentration only. For the W1PR1 and W1PR2 cells, we did not observe any significant influence of piperine on cell migration (Figure 14A,B).

## 3. Discussion

Ovarian cancer is responsible for 5% of all cancer-related mortality in women [57]. Despite therapeutic improvements in ovarian cancer treatment, its mortality rate is still high. Late diagnosis and development of drug resistance are the main reasons for treatment failure in epithelial ovarian cancer (EOC) [3].

The presence of CSCs [26,29,30,31,32,33,34,58], expression of drug transporters [11,13,31,58], and ECM molecules [18,19,20,21,22,23,24,27,28] are the most important mechanism of drug resistance. Increased expression of drug resistance genes seems to be associated with increased signal transduction that results from an imbalance between phosphorylation and dephosphorylation of tyrosine [35,36,37,38,39,40,59].

An increasing number of studies have discovered that many plant-derived molecules with anticancer effects can be considered potential therapeutic drugs in cancer treatment. Moreover, plant-derived molecules are believed to be safe for humans, as they show low or even no adverse effects on the human body [46]. Piperine has been suggested to possess potential anticancer effects in various malignant cancers [60,61,62]. Thus, we were interested if piperine can influence drug resistance in ovarian cancer cell lines.

First, we compared the sensitivity of investigated cell lines to piperine. Surprisingly, both PAC-resistant cell lines were much more sensitive to piperine than the parental W1 cell line. It indicates that piperine or its derivatives may be a good candidate as an agent that can break down chemotherapy resistance in some drug-resistant cancers. The cytotoxic effect of piperine has also been described in the A2780 ovarian cancer cell line [45]. Based on that information, we checked if piperine can increase the cytotoxic effect of anticancer drugs. We observed higher sensitivity to PAC or TOP after cotreatment with piperine in PAC- and TOP-resistant cell lines. A synergistic effect of PAC and piperine was also observed in the MCF-7 breast cancer cell line [48], in HER2-overexpressing breast cancer cells [47], and in the SKOV-3 ovarian cancer cell line [49]. However, we did not find any literature data concerning the breakdown of TOP resistance by piperine. Thus, our observation is first in the world. The obtained results suggest that piperine can be considered a potential agent enhancing the effectiveness of chemotherapy.

Our study’s next step was devoted to the mechanism responsible for piperine action on drug-resistant cells. Previously, we reported a downregulation of PTPRK expression in 15 drug-resistant ovarian cancer cell lines derived from W1 and A2780 ovarian cancer cell lines [40]. Loss of PTPRK expression or function was also associated with increased chemotherapy resistance in NKTCL patients [63]. In contrast, PTPRK overexpression was followed by higher sensitivity to cytotoxic drugs in acute lymphoblastic leukemia (ALL) cell lines [54] and glioma cells [39]. Our results show that loss of PTPRK expression was associated with a strong increase in total pTYR levels [40], suggesting that a decrease in PTPRK expression led to enhanced signaling pathways activity. It seems to be supported by others, as a restoration of PTPRK expression in cancer cell lines resulted in a reduction of phosphorylated extracellular signal-regulated kinases 1 and 2 (Erk 1/2), protein kinase B, (Akt), STAT3, and STAT5 [54]. After piperine treatment, we observed the restoration of PTPRK expression and decrease in total pTYR level in investigated cell lines, suggesting that signaling pathways involving tyrosine phosphorylation are among the main targets of piperine action. Others made similar observations. A prostate cancer cell line study revealed that piperine decreased the expression of phosphorylated STAT-3 and nuclear factor-κB (NF-κB) [54]. Inhibition of ERK1/2, p38 MAPK, and Akt signaling pathways in breast cancer cell line [47] and kinase C and ERK1/2 in fibrosarcoma cells by piperine [64] has also been reported. In summary, loss of PTPRK expression and increased signal transduction activity seems to be a primary drug resistance development mechanism in investigated cell lines, which is responsible for an increased expression of drug-resistant genes. In contrast, the restoration of PTPRK expression and decrease in pTYR level can be a primary reason for the chemoresistance loss after piperine treatment.

According to the CSCs model of drug resistance development, CSCs are responsible for developing drug resistance in cancer [33]. One of the universal markers of CSCs among solid tumors is the expression of ALDH1A1 [33], and the presence of ALDH1A1 cells correlated with ovarian cancer progression [65,66] and drug resistance [65,66,67]. It has been reported that all three CSCs signaling pathways (Wnt/β-catenin, Hedgehog, and Notch) can be a target for piperine [68]. In breast cancer, piperine inhibited the Wnt/β-catenin pathway and reduced the number of ALDH1+ cells [52]. We observed ALDH1A1+ cells population in W1PR1, W1PR2, and W1TR cell lines [21,34]. Thus, we were interested if piperine can target ALDH1A1 cells in investigated cell lines. In W1PR1 cell line we observed minimal effect of piperine on ALDH1A1 expression. In contrast, a strong downregulation of ALDH1A1 expression and reduction of ALDH1A1+ cell numbers were observed in W1PR2 and W1TR cell lines. It indicates that CSCs can also be a target for piperine in ovarian cancer, and a reduction of CSCs in tumors can increase the effectiveness of chemotherapy.

It has been suggested that piperine can influence drug transporters’ activity and expression, resulting in increased sensitivity to anticancer drugs [50,51,69]. In the MCF-7/DOX cell line, piperine treatment decreased the activity and expression of P-gp/MDR1 and BCRP and increased sensitivity to DOX and mitoxantrone [49]. An in vivo study on ICR-NOD/SCID mice showed that piperine significantly increased the antitumor effect of docetaxel on taxane-resistant prostate cancer and decreased *MDR1* gene expression in the MDCK cell line [50]. Thus, we were interested if piperine can influence drug transporters expression in investigated cell lines. We observed a low to moderate decrease in P-gp expression in both PAC-resistant cell lines and a strong downregulation of BCRP expression in TOP-resistant cell lines. Thus, another probable reason of higher sensitivity to chemotherapeutic drugs after piperine treatment can be the downregulation of drug transporters activity and/or expression. The other question is if piperine directly or indirectly may regulate drug transporters’ activity and/or expression. It has been observed that piperine directly binds to ABC proteins, decreasing its ATP-aze activity [69]. On the other hand, others suggest that drug transporters’ activity is regulated by phosphorylation [70], and a direct role of phosphorylation in BCRP protein dimerization and activity has been described [71]. As the downregulation of *MDR1*, *MRP1,* and *BCRP* genes after piperine treatment has been reported [49,50], and we observed a correlation between reduced pTYR level with the downregulation of drug transporters expression after piperine treatment, it is also possible that the downregulation of these genes and/or activity can result from decreased signaling pathways activity.

Another drug resistance development model postulates that cancer cells overexpressing extracellular matrix proteins (ECM) are more resistant to chemotherapy [19]. Recently, we observed very high upregulation of different collagen genes/proteins in ovarian cancer cell lines [27]. COL3A1 was overexpressed in the W1PR1 cell line and much more in W1PR2 and W1TR cell lines [27]. COL3A1 was present not only as a cellular protein inside the cancer cells but also in cell culture medium as a secreted protein [27]. Furthermore, we observed different populations of cells with very high, low, and lack of COL3A1 expression, and the highest COL3A1 expression was observed in ALDH1A1+ cells [23,27]. The direct binding of cytostatic drugs such as DOX, MTX, and PAC to ECM molecules limits their ability to tumor tissue [17]. We also considered this in our model. The other possibility assumes drug diffusion blocking by extracellular as well as intracellular collagen. In many drug-resistant tumors with a high level of ECM expression, the time required for drug penetration is lengthened, resulting in lower drug concentration in the tumor and drug resistance [16,72,73]. Finally, the interaction of cancer cells by cell surface receptors (mainly integrins) with ECM components increase signal transduction, resulting in an increased expression of drug resistance genes and resistance to apoptosis [20,74]. The expression of COL3A1 was also observed in ovarian cancer patients and was related to CIS resistance [75]. The above expression of COL3A1 in drug-resistant cell lines seems to be an important mechanism of drug resistance. After piperine treatment, we observed a significant decrease in fluorescence signal in all three cell lines. Mostly, a reduced number of cells with very high expression of COL3A1 was noted and correlated with a decreased expression of COL3A1 in cell lysates of W1PR2 and W1TR cell lines. We hypothesized that a decreased level of extracellular COL3A1 resulted in reduced drug binding, increased drug diffusion into cells, and reduced interaction between ECM and cell surface receptors, resulting in reduced CAM-DR strength. It can result in increased sensitivity to TOP after piperine treatment. On the contrary, the W1PR2 cell line revealed an increased level of extracellular COL3A1 after piperine treatment that may strengthen the CAM-DR mechanism and lower PAC sensitivity. Indeed, in the W1PR2 cell line, we observed a minimal synergistic effect of piperine on PAC resistance that may be explained by the protective effect of extracellular COL3A1 in the way of CAM-DR.

One more aspect of drug resistance should be considered. In all resistant cell lines, piperine treatment leads to increased PTPRK expression. PTPRK can influence CAM-DR by dephosphorization of the AKT and MAPK pathways [53]. Thus, the re-expression of PTPRK after piperine treatment can result in decreased CAM-DR. In the W1PR2 cell line, the strength of CAM-DR can result from dephosphorization signaling molecules by PTPRK and stimulation of CAM-DR by extracellular COL3A1. We looked through the literature data, but we did not find any relation between piperine and COL3A1 or other ECM components. Thus, the regulation of COL3A1 expression by piperine is a new observation.

**Figure 15:**
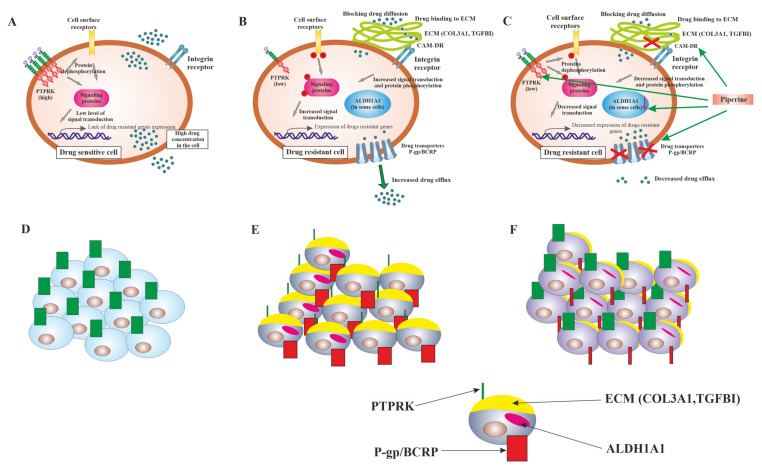
Main mechanisms of piperine action on drug-resistant cells. Drug-sensitive cells (W1) are characterized by a high expression level of protein tyrosine phosphatase kappa (PTPRK), resulting in dephosphorylation of cell surface receptors and other signaling proteins. It eventually leads to low (if any) expression of drug-resistant genes and high drug concentration in the cell (**A**). In those cells, the expression of drug transporters and ECM molecules is not observed (**D**). In drug-resistant cells, we observed a decreased level of PTPRK expression, resulting in an increased total level of pTYR in the cell and probably increased signal transduction, leading to an increased expression of drug-resistant genes. Drug-resistant cells are characterized by a high level of drug transporters expression (P-gp or BCRP) and a high level of ECM molecules (COL3A1) expression (**B**,**E**). On the one hand, drug transporters decrease drug concentration in the cell below therapeutic concentration. On the other, a very high level of COL3A1 can directly bind cytotoxic drugs and block drug diffusion inside the cancer cell. Additionally, a high level of extracellular collagen can bind cell surface receptors, such as integrin receptors, and stimulate CAM-DR. Some cancer cells are also characterized by a high level of cancer stem cells marker–ALDH1A1 (**B**,**E**). The piperine treatment of drug-resistant cells resulted in an increase in PTPRK expression and decrease in pTYR level, resulting in decreased signal transduction and decreased expression of drug-resistant genes. Piperine treatment also decreases drug transporters’ (P-gp/BCRP) level or activity, resulting in higher drug concentration in the cell. Decreased ECMs levels (COL3A1, TGFBI) lead to higher drug concentration in the cell and decreased CAM-DR. The number of ALDH1A1 positive cells and/or expression of ALDH1A1 decrease (**C**,**F**). All these events lead to higher sensitivity to cytotoxic drugs.

TGFBI (transforming growth factor-beta-induced protein) is an ECM secretory protein with dual function in ovarian cancer [76]. In most papers, the expression of TGFBI is silenced in ovarian cancer tissue [77] and cell lines [78], and it correlates with PAC resistance [78,79]. On the other hand, TGFBI promoted invasion and metastasis of ovarian cancer [76]. Patients with high tumor expression of TGFBI have significantly shorter OS [80]. Previously, we observed an upregulation of TGFBI in three TOP-resistant ovarian cancer cell lines and suggested its role as one of the TOP-resistance genes [55]. Thus, in this study, we examined the impact of piperine on TGFBI expression in a TOP-resistant cell line, but no such influence was noted. However, a significant reduction in TGFBI secretion after piperine treatment was observed. TGFBI is a protein inducible by TGFβ1 and secreted by different types of cells. It is a part of ECM that binds to collagen and interacts with integrins on cell surfaces, leading to activation of different signaling pathways and cancer progression. In glioma cells, a high TGFBI expression was associated with poor prognosis and phosphorylation of AKT and mTOR [81]. An upregulation of TGFBI was observed in gastrointestinal tract cancers and resulted in activation of the FAK/AKT/AKT1S1/PRS6/EIF4EBP pathway, playing a role in cell survival and proliferation [82]. In pancreatic cancer, TGFBI stimulated the FAK signaling pathway by binding to integrin αVβ5 [83]. Thus, the limitation of TGFBI secretion by piperine can inhibit integrin signaling, which is the primary signaling in CAM-DR, and it eventually leads to higher TOP sensitivity [84]. Figure 15 summarizes the main mechanisms of piperine action on drug-resistant cells in our model.

Another aspect of piperine effect on tumor cells involves an inhibition of cancer cell proliferation and migration. It has been reported that piperine can arrest the cell cycle at different phases via the induction and inhibition of various protein regulators and checkpoints. However, this effect can be cell type-dependent. In the OVCAR-3 ovarian cancer cell line, piperine exerted antiproliferative effects by apoptotic cell death, induced arrest of cell cycle at the G2/M phase, and blocking the PI3K/Akt/GSK3β signal transduction pathway [46]. In another ovarian cancer cell line A2780, piperine suppressed cell proliferation by induction of the intrinsic apoptotic pathway [45]. Here, we also observe an apparent inhibitory effect on cell proliferation. However, we did not investigate the mechanism responsible for piperine action in our model.

The antimigratory effect of piperine in a concentration-dependent manner was observed in the OVCAR-3 cell line [46]. In the DU145 prostate cancer cell line, piperine markedly reduced cell migration [41]. Inhibition of the Wnt/β-catenin signaling pathway and cell migration was observed in colorectal cancer cells [85]. In another colorectal cancer study, piperine inhibited the migration and invasion of cancer cells, reversed the epithelial-to-mesenchymal transition biomarker expression [86], and downregulated STAT3 expression. In contrast to this study, we did not observe any effect of piperine on the migration of PAC-resistant cell lines. However, an inhibitory effect for IC50 concentration in the TOP-resistant cell line was observed. The differential effect of piperine on cell migration may be due to several reasons. First, in our experiment, there is a difference in IC50 between PAC- (19 µM for W1PR1 and 9.7 µM for W1PR2) and TOP-resistant cell line (93 µM). Secondly, PAC and TOP target cancer cells by different mechanisms: inhibition of microtubule polymerization [87] or topoisomerase I [88,89], respectively. This is why we assume that these cell lines cells developed not only different drug resistance mechanisms but probably also different expression and/or activity of proteins/pathways responsible for cell migration that can differ in sensitivity to piperine. Explanation of the differences in the effect of piperine on cell migration requires a detailed study of the migratory pathways.

Our study presents a pleiotropic effect of piperine on different drug resistance mechanism in ovarian cancer model of drug resistance development. The results are promising and may indicate where to focus in research on the piperine cellular targets in drug-resistant cancer cells. 

## 4. Material and Methods

### 4.1. Reagents and Antibodies

Culture media (RPMI-1640), fetal bovine serum, antibiotic–antimycotic solution, L-glutamine, DAPI mounting medium, and PAC and TOP were purchased from Sigma (Sigma-Aldrich, Poznan, Poland). Rabbit monoclonal anti-ALDH1A1 Ab was purchased from Abcam (Abcam, Cambridge, UK), mouse monoclonal anti-COL3A1 was purchased from Invitrogen (Invitrogen, Carlsbad, CA, USA), mouse monoclonal anti-PTPRK Ab, anti-p-Tyr, mouse monoclonal anti-MYOT Ab (B-3), and rabbit polyclonal anti-GADPH Ab were obtained from Santa Cruz Biotechnology (Santa Cruz, CA, USA). Mouse monoclonal anti-P-gp Ab was purchased from Invitrogen (Thermo Fisher Scientific, Waltham, MA, USA). Rabbit polyclonal anti-TGFBI was purchased from Atlas Antibodies (Stockholm, Sweden). Mouse anti-Ki67 was purchased from Dako (Glostrup, Denmark). Donkey anti-goat horseradish peroxidase (HRP)-conjugated Ab was purchased from Santa Cruz Biotechnology (Santa Cruz Biotechnology Inc., Dallas, TX, USA). The fluorescent MFP488 donkey anti-goat IgG was obtained from MoBiTec (MoBiTec, Molecular Biotechnology, Goettingen, Germany) and fluorescent Alexa Fluor^®^488 and Alexa Fluor^®^594 Donkey Anti-Rabbit IgG from Jackson ImmunoResearch Laboratories (Jackson ImmunoResearch Laboratories, Cambridgeshire, UK). Western blot reagents (membranes, gels and protein marker) were purchased from Bio-Rad (Bio-Rad Laboratories Ltd., Watford, Hertfirdshire, UK).

### 4.2. Cell Culture

The human primary ovarian cancer cell line W1 was established from the tumor tissue of an untreated 54-year-old Caucasian female patient diagnosed with serous ovarian adenocarcinoma (G3, FIGO IIIc). Cells grow as a monolayer and present an epithelial morphology and adherent growth model described previously [21]. Sublines resistant to PAC (W1PR1 and W1PR2) and TOP (W1TR) were derived by exposure of the W1 line to incrementally increasing concentrations of relevant drug. The final concentration of PAC was 1100 ng/mL, and the final concentration of TOP was 24 ng/mL. The cells were grown in RPMI medium supplemented with 10% FBS, antibiotic/antimitotic solution, 1% L-glutamine at 37 °C in a humidified atmosphere and 5% CO2. The resistant cell lines W1PR1 and W1PR2 were grown in culture media containing 1100 ng/mL of PAC, and W1TR was grown in culture media containing 24 ng/mL of TOP in order to maintain the resistance. The increase in resistance according to parental drug-sensitive cell line W1 was as follows: 20-fold for W1TR vs. W1; 641-fold for W1PR1 vs. W1 and 967-fold for W1PR2 vs. W1 as described previously [90].

### 4.3. Cell Viability Assay

The cell viability was evaluated using MTT assay. In the first step, we determined the IC25 and IC50 concentration of piperine. For this purpose, parental cells were seeded at a density of 4 × 10^3^ cells/well in a 96-well plate, but resistant cell lines were seeded at a density of 7 × 10^3^ cells/well and incubated for 48 h to retain their morphology. After that time, the cells were treated with fresh medium supplemented with or without increasing concentrations of piperine and incubated for 72 h at 37 °C. After 72 h of exposure, 10 µL of the MTT labeling reagent was added to the medium (the final concentration of MTT was 0.5 mg/mL), and the cells were incubated for additional 4 h. Following this process, 100 µL of solubilization solution was added to each well. The absorbance of each sample was measured in a microplate reader at 570 nm with a reference wavelength of 720 nm, according to the manufacturer’s protocol. The negative control was conducted using cell-free culture medium containing both the MTT reagent and solubilization solution. The experiments were repeated three times, and each concentration in a given experiment was tested in duplicates. Cell viability was expressed as a percentage of the untreated control (means ± SEM).

Next, cells were incubated with PAC/TOP or piperine alone or in combination of PAC/TOP with piperine for 72 h at 37 °C in a humidified atmosphere and 5% CO_2_. The effect on cells viability was analyzed in similar way as described before. The proportion of cell survival (%) was calculated using the formula (OD of drug treated sample-blank)/(OD of control-blank) × 100%.

### 4.4. Immunofluorescence

Cells were seeded onto the coverslips and incubated for 24 h to attach. After that time, the cells were treated with a fresh medium supplemented with or without increasing concentrations of piperine and incubated for 72 h at 37 °C. Cells were fixed and permeabilized with ice-cold acetone/methanol (1:1) for 10 min. Cells were washed three times, five minutes each in PBS, and then blocked with 3% BSA in PBST for 30 min at room temperature. Later, cells were incubated in primary antibody solution against: P-gp (mouse anti-P-gp antibody, 1:200), Ki67 (mouse anti-Ki67 antibody, 1:200), PRPRK (mouse anti-PTPRK antibody, 1:100), pTYR (mouse anti-pTYR antibody, 1:100), COL3A1 (goat anti-COL3A1 antibody, 1:100), ALDH1A1 (rabbit anti-ALDH1A1 antibody, 1:100), BCRP (rabbit anti-BCRP antibody, 1:300), and TGFBI (rabbit anti-TGFBI antibody, 1:500) for 2 h at room temperature. Subsequently, cells were washed 3 times, five minutes each in PBS, and incubated with respective green dye-labeled secondary antibodies for 1 h at room temperature (Alexa Fluor^®^488, donkey Anti-Mouse or Anti-Rabbit IgG IgG, 1:200, Jackson ImmunoResearch Laboratories, Cambridgeshire, UK; MFP488, donkey anti-goat IgG, 1:200, MoBiTec, Goettingen, Germany). Finally, cells were washed 3 times, five minutes each in PBS, and mounted in DAPI mounting medium. Images were acquired using fluorescence microscope (Zeiss Axio-Imager.Z1).

### 4.5. Protein Isolation and Western Blot

Cells were seeded into the cell culture bottles for 48 h to attach. After that time, the cells were treated with fresh medium supplemented with or without increasing concentrations of piperine and incubated for 72 h at 37 °C. The cells (1 × 10^6^ cells/25 µL lysis buffer) were lysed with ice-cold RIPA buffer containing protease inhibitors (Roche Diagnostics GmbH, Mannheim, Germany). Then, the lysate was centrifuged at 12,000 rpm at 4 °C for 15 min, and the supernatant was collected. The isolation of proteins from culture media was prepared after a 72 h culture of cells in serum-free media. Next, the media was centrifuged at 15,000 rpm for 30 min at RT, and supernatants were placed in Amicon Ultra-15 3K centrifuge filter devices (Sigma, St. Louis, MO, USA) and centrifuged using a swinging-bucket rotor for 60 min at 4000× *g*/*RT*. The protein concentration was determined by the Bradford method (Bio-Rad Laboratories, Hemel Hempstead, UK). Then, a 25 µg protein sample was loaded, separated on gradient a 4–20% mini-PROTEAN^®^ TGX™ precast gel using the SDS-PAGE electrophoresis, and transferred onto nitrocellulose membrane. The membrane was blocked with 5% non-fat milk for 1 h at room temperature and probed using primary antibodies against ALDH1A1/BCRP at a 1:500 dilution, P-gp (1:1000), TGFBI/COL3A1 at a 1:500 dilution also, overnight at 4 °C and followed by incubation with HRP-labeled secondary antibodies. Signals were developed using a chemiluminescence detection system (ECL, Femto Super Signal Reagent) and Hyperfilm ECL (GE Healthcare, Buckinghamshire, UK). The protein loading was normalized by reblotting the membranes with rabbit anti-GADPH Ab (Santa Cruz Biotechnology), at a 1:1000 dilution and goat anti-rabbit HRP-conjugated Ab (Santa Cruz Biotechnology). The relative density of investigated proteins to that of GADPH was analyzed with ImageJ Java-based image processing program developed at the National Institutes of Health (University of Wisconsin, Madison, WI, USA).

### 4.6. Proliferation Assay

For the cell proliferation assay, 2 × 10^5^ cells/well were seeded in a 6-well plate and incubated at 37 °C and 5% CO_2_ for 24 h. After this, the cells were incubated with piperine for 72 h. After the treatment, the cells were washed with PBS, trypsinized, and counted with Trypan Blue. All of the experiments were performed in triplicate, at least.

### 4.7. Migration Assay/Wound Healing Assay

The W1PR1, W1PR2, and W1TR cells were seeded in 6-well plates and cultured for 48 h to allow them to reach 80–90% confluence. Then, the cells were scraped carefully using a sterile 200-μL plastic pipette tip to make a scratch. Debris was removed from the cultures, and the cells were treated with piperine. The width of the denuded area was assessed at 0, 24, and 48 h using a phase contrast microscope. The migration rate was calculated using the following equation: migration rate = (average original width − average final width)/average original width × 100%.

### 4.8. Statistical Analysis

All data obtained in the experiments were analyzed using Student’s *t*-test. The statistical significance interval was determined at *p* < 0.05.

## 5. Conclusions

This study investigated the effect of piperine treatment on PAC- and TOP-resistant ovarian cancer cell lines. The difference in sensitivity to piperine, observed among investigated cell lines, indicates that even drug-resistant cancers may be sensitive to piperine in monotherapy. Furthermore, piperine enhanced the cytotoxic effect of chemotherapeutic agents in all drug-resistant cell lines. Piperine seems to target different drug-resistance mechanisms in cancer cells. As we observed, reduced protein phosphorylation resulting probably in reduced signal transduction, reduced number of CSCs, decreased expression of drug transporters, and decreased expression of ECM molecules, resulting likely in reduced CAM-DR. It may indicate that piperine or its derivatives should be considered a potential anticancer agent that could improve chemotherapy’s effectiveness in cancer patients.

## Figures and Tables

**Table 1:** Summary of cell lines resistance to piperine. ** *p* < 0.01.

Cell Line	Piperine IC25 [µM]	Piperine IC50 [µM]
W1	64	106
	(57–71)	(96–115)
	1	1
W1TR	76	93
	(75–79)	(88–95)
	1.19 ↑	1.14 ↓
W1PR1	9.5	19
	(7.6–12.5)	(15–23)
	6.7↓ **	5.6 ↓ **
W1PR2	4.4	9.7
	(2.5–5.8)	(8.0–12.1)
	14.5 ↓ **	10.9 ↓ **

The piperine IC25 and IC50 are indicated for each cell line. The piperine resistance in the parental W1 cell line was assigned a value of 1. Underlined values indicate multiplicities of resistance with respect to the W1 cell line. The up/down arrows indicate increase or decrease in IC25 or IC50 compared to the W1 cell line. ** *p* < 0.01.

## Data Availability

Not applicable.
